# Transmission of measles among healthcare Workers in Hospital W, Xinjiang Autonomous Region, China, 2016

**DOI:** 10.1186/s12879-018-2950-y

**Published:** 2018-01-12

**Authors:** Haimei Jia, Chao Ma, Mengting Lu, Jianping Fu, Lance E. Rodewald, Qiru Su, Huaqin Wang, Lixin Hao

**Affiliations:** 1 Fuzhou Center for Disease Control and Prevention, No.95, Quanzhong Road, Fuzhou, Fujian 350004 China; 2 Chinese Center for Disease Control and Prevention, No.27, Nanwei Road, Xicheng District, Beijing, 100050 China; 3 The Center for Disease Control and Prevention of Xinjiang Production and Construction Corps, Urumchi, Xinjiang Autonomous Region 830002 China; 4 Office of the WHO Representative in the People’s Republic of China, Beijing, 100600 China

**Keywords:** Measles, Healthcare workers, Outbreak, Nosocomial transmission, Vaccination

## Abstract

**Background:**

As China approaches the elimination of measles, outbreaks of measles continue to occur. Healthcare workers (HCWs) are known to be at high risk of infection and transmission of measles virus. A measles outbreak occurred in a hospital in Xinjiang Uighur Autonomous Region of the People’s Republic of China. We report an investigation of this outbreak and its implications for measles elimination and outbreak preparedness.

**Methods:**

We conducted a retrospective search for measles cases using hospital records. Information on cases was collected by interview, and was used to determine epidemiological linkages. We surveyed HCWs to determine their demographic characteristics, disease history and vaccination status, and knowledge about measles.

**Results:**

We identified 19 cases, ages 18 to 45 years, in Hospital W between December 2015 and January 2016; 14 were laboratory-confirmed, and 5 were epidemiologically linked. The primary case was a 25-year-old neurology department nurse who developed a rash on 22 December 2015 that was reported on 11 January 2016. She continued working and living with her workmates in a dormitory during her measles transmission period. Among the 19 infected HCWs, 2 had received a dose of measles-containing vaccine (MCV) before the outbreak, and 16 had unknown vaccination status. Outbreak response immunization activities were started on 8 January in a non-selective manner by offering vaccine regardless of vaccination history; 605(68%) of 890 HCWs were vaccinated. The HCW survey had a 73% response rate (646/890); 41% of HCWs reported that they had received MCV before outbreak, and 56% exhibited good knowledge of measles symptoms, transmission, complications, and vaccination.

**Conclusions:**

Low MCV coverage, low measles knowledge among HCWs, delayed reporting of measles cases, and absence of proper case management were associated with this outbreak. Training and vaccinating HCWs against measles are essential activities to prevent measles virus transmission among HCWs.

## Introduction

Measles is a highly-contagious, acute illness that can transmit measles virus to 75–90% of susceptible contacts [1]. In 2006, China endorsed the 2006–2012 National Action Plan for Measles Elimination, which continued a two-dose measles vaccination strategy (administered at 8 months and 18–23 months) and called for routine measles vaccine coverage to be greater than 95% for both measles vaccine doses in every county. The Action Plan called for conducting supplementary immunization activities (SIAs) to close immunity gaps among children and strengthening laboratory-supported surveillance. During implementation of the Action Plan, the incidence of measles decreased substantially, from 99.4 per million population in 2008 to 4.6 per million in 2012 [2].

As China approaches measles elimination, indigenous-strain measles virus outbreaks continue to occur among groups of susceptible individuals. Health care workers (HCWs) caring for measles patients have frequent face-to-face contact with infectious individuals, placing non-immune HCWs at high risk of infection. HCWs who become infected have the potential to transmit measles virus to other hospital staff and to patients, some of which may be vulnerable to severe illness and complications from measles. In January 2016, Hospital W in Xinjiang Autonomous Region of western China reported measles cases among staff. We describe the investigation and response of this outbreak, which highlights the importance of vaccinating HCWs and preparing appropriately for measles outbreak prevention and control.

## Methods

### Setting

Hospital W is a general hospital with 890 staff in 58 departments including Emergency, Pediatrics, Internal Medicine, and other departments; it is located in northern Xinjiang Autonomous Region, which has high measles vaccination coverage (≥95%) among age-eligible children. Between 2011 and the start of this outbreak, there had been only 6 HCW measles cases reported in Hospital W.

### Case identification

We conducted a retrospective search for measles cases among Hospital W HCWs. A suspected case was defined as a person with fever (≥ 37.5°C) plus rash or conjunctivitis, or an individual suspected by a physician in Hospital W to have measles, with onset between December 2015 and February 2016. Based on Chinese Measles Surveillance Guidelines [3], which are consistent with WHO guidelines, suspected cases were classified as laboratory-confirmed measles cases, epidemiologically-linked and confirmed measles cases, clinically-diagnosed measles cases, or discarded cases. We considered the measles incubation period to be 7 to 21 days before rash onset.

Case investigation forms were completed and blood specimens or throat swab were collected from suspected cases in accordance with Chinese Measles Surveillance Guidelines [3]. Measles cases are required to be reported to the China’s National Notifiable Disease Reporting System (NNDRS). The investigators attempted to identify all measles cases through a combination of this passive surveillance system and through active search that included Hospital W medical records, HCWs work schedules, and interviews of all HCW cases. We attempted to obtain the following information about each case: demographic data, illness onset, vaccination history, history of contact with measles cases, travel history during the incubation period, and activities 5 days before and after rash onset. Laboratory tests were conducted by the Wujiaqu County Centers for Disease Control and Prevention (WJQ CDC).

### Staff survey

We conducted a survey targeting all staff using a standardized, self-administered questionnaire to obtain information on demographic characteristics (sex, age, department, and duration of employment), history of measles, attitudes and knowledge toward measles and measles vaccination, and self-reported vaccination status.

## Results

Between December 2015 and January 2016, we confirmed 19 of 20 suspected cases among Hospital W HCWs. The age-range of confirmed cases was18 to 45 years; 14 cases were laboratory-confirmed and 5 were epidemiologically-linked; 1 suspected case was determined to not be measles. The attack rate was 2.13%; no serious cases occurred. We were unable to find any cases among Hospital W patients or among family members of cases.

### Primary case

On December 18, 2015, a 25-year-old neurology nurse of Hospital W (patient 1) felt ill and stayed away from work for 2 days; she developed a rash 4 days later. Intravenous antibiotics had been administered prior to rash onset; she worked occasionally at the hospital after onset of the rash. She was initially suspected of having an allergic rash, but on January 10, she was recognized as possibly having measles and was placed in respiratory isolation in Hospital W. She was reported to NNDRS as being suspected to have measles, and blood was drawn for confirmatory testing by WJQ CDC. On January 11, laboratory testing showed that she was positive for measles immunoglobulin M (IgM).This individual had been living in the staff dormitory with her workmates, and she had had lunch in the staff dining hall during her measles transmission-period. During the time of her illness, other HCWs became ill. Her recent travel history showed that she attended a wedding in F country on December 12, 2015, 10 days prior to rash onset, where measles cases had been reported in December.

### Outbreak description

**Fig. 1:**
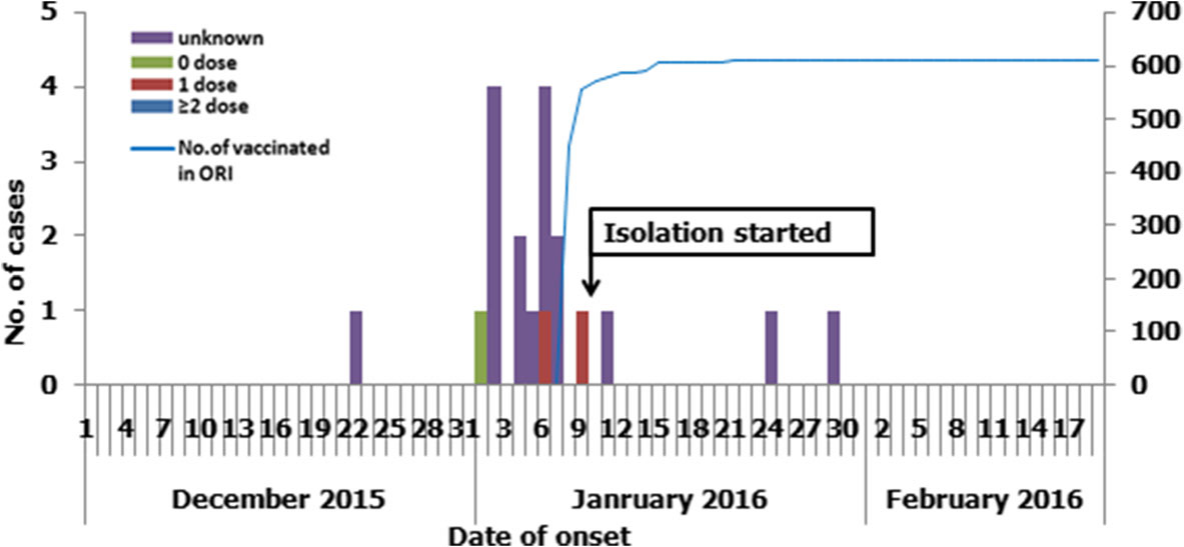
Confirmed measles cases by illness onset, December 2015 through January 2016. Shown is the time course of the epidemic with the vaccination status of the 19 cases of measles, all hospital staff, the number of hospital staff vaccinated (out of 890 staff), and implementation of isolation measures

**Fig. 2:**
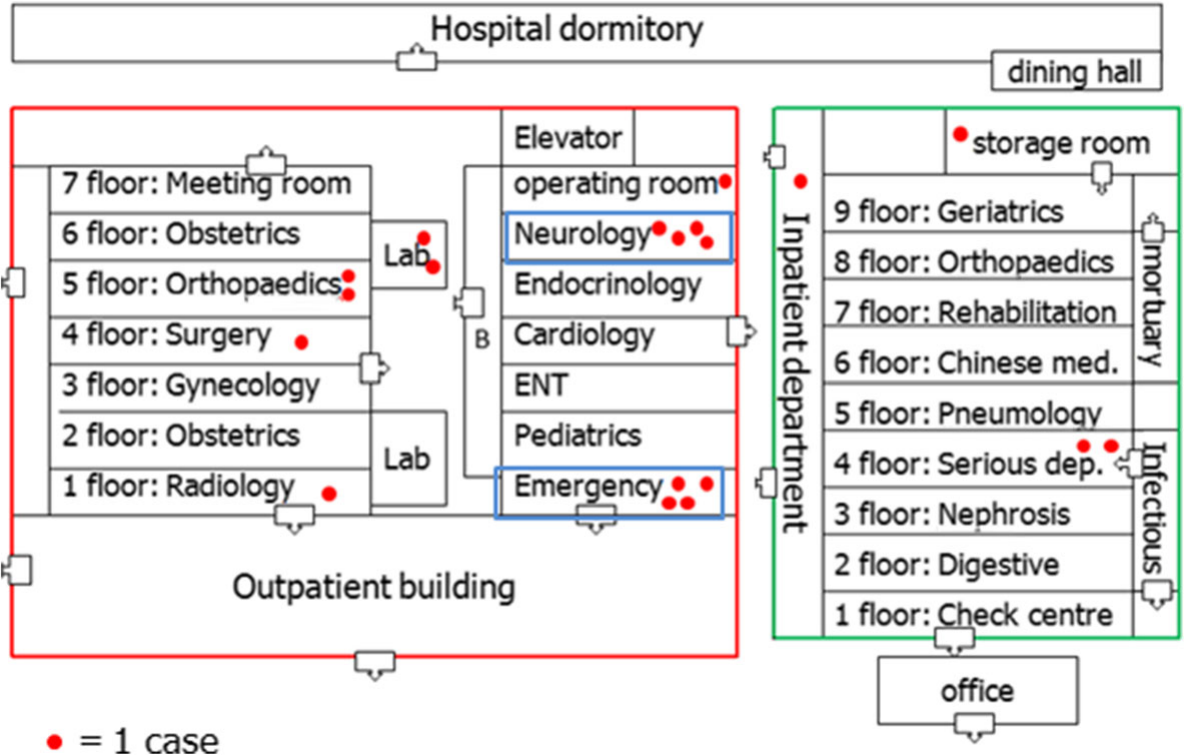
Hospital W layout, and distribution of the 19 measles cases by department

Figure 1 shows the time course of the outbreak and the vaccination status of cases. A total of 19 individuals, all hospital staff, were identified to have acquired measles between the onset of the primary case and January 29, 2016, the illness onset of the last case. The male to female ratio in the outbreak was 0.9:1; the median age of cases was 26 years, with an age range of 18 to 45 years. The number of confirmed measles cases reached a peak during the second week of the outbreak. Figure 2 shows the distribution of cases by hospital department - staff in 8 different departments acquired measles. Of the 19 cases, the vaccination status was unknown for 18 (84.2%); 2 individuals received one dose of MCV; 1 individual was documented to have never received MCV.

**Fig. 3:**
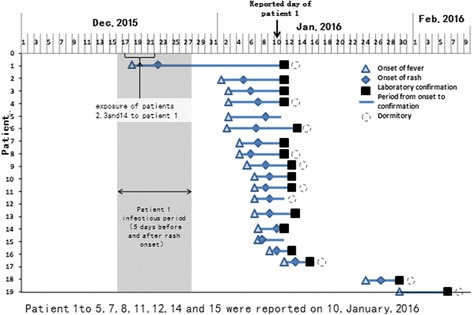
Case-by-case timing of fever onset, rash onset, and laboratory confirmation of measles. The median time from onset to diagnosis was 7 days (range 3–24)

The primary case developed a rash on December 22, 2015 and was reported through NNDRS 20 days later, on January 10, 2016, at which time12 other HCWs cases were also reported. The median time from onset to diagnosis was 7 days (range 3–24). Figure 3 shows the time and generational distribution of cases.

The primary case’s workmates (patients 2 and 3), both nurses working in Neurology, had onset of rash on January 5. The other 9 HCW cases worked in different departments and had rash onset between January 6 and 10; these individuals lived with the primary casein a staff dormitory and dined together in the staff dining hall. These 12 cases all tested positive for measles IgM, triggering their reports to NNDRS. Five additional cases had rash onset between January 7 and 11 and were reported to NNDRS on January 13. The last two cases in the outbreak were doctors in the Surgery Department and the Emergency Department; their rash onsets were January 26 and 29, respectively, and sera tested positive for measles IgM on January 29 and February 6.

Our investigation showed that 19 confirmed cases had been infected through hospital acquired transmission or exposure in the hospital living spaces. Figures 1 and 3 illustrates 3 generations of transmission that lasted 5 weeks.

### Outbreak response

On January 8, the hospital began emergency measles vaccination, targeting all HCWs, regardless of vaccination history. On the first day of the emergency vaccination, 107 HCWs were vaccinated, and by January 21, 608 HCWs had been vaccinated (Fig. 1). On January 10, the hospital implemented strict isolation measures, including using a special ward to receive suspected measles cases and distributing masks for respiratory precautions. Surveillance for measles was intensified, and WJQ CDC worked with the hospital to identify and test suspected cases. The hospital monitored close contacts’ temperatures for 21 days after their last contact with a measles case, and reported individuals with fever or rash cases to the Infectious Diseases Department.

### HCW survey

Of the 890 Hospital W HCWs, 646 (72.6%) returned survey questionnaires. The mean age of the responding HCWs was 29 years (range 18–60); 464 (71.8%) were female; 41.3% (266) were nurses; 37.2% (239) were doctors; and 21.5% (139) were other types of HCWs. Among respondents, 268 (41.2%) knew that they had not been immunized, while261 (40.4%) reported a history of 1 MCV dose, and 19 (2.9%) reported receiving two MCV doses.

The questionnaire asked about measles and measles vaccination: 62.5% knew the symptoms of measles; 89.2% knew how measles is transmitted; 54.5% knew the complications of measles; 31.0% agreed that measles vaccine is safe, and 36.1% agreed that measles vaccine is effective. When asked about willingness to get vaccinated, 34.5% said they were willing and the other HCWs said they were not willing to get vaccinated, and among those unwilling to get vaccinated, 45.7% stated that they did not care about the risk of infection.

## Discussion

We have described a 19-case hospital-based measles outbreak characterized by 3generations of transmission, that was limited to health care workers, and that stopped after a measles vaccination campaign targeting hospital staff. The outbreak was started by a nurse who was likely infected while attending a wedding; she continued to work while ill and continued to reside in a dormitory with other hospital staff. The cause of her illness was initially misdiagnosed. Vaccination coverage prior to the outbreak was low among the health care workers, and their knowledge about measles and their confidence in the safety and effectiveness of measles vaccine were also low. Delays in diagnosis and reporting delayed proper management of the outbreak.

Measles is a highly contagious infectious disease that can be readily transmitted among susceptible individuals in close quarters such as health care facilities [4]. In this outbreak, the primary case was suspected of having an allergic rash, and 20 days later, she was recognized as possibly having measles and was placed in respiratory isolation in Hospital W. During her illness other HCWs became ill. The initial case in this outbreak likely brought measles to the hospital, but often an ill patient is the source of a nosocomial outbreak. Of interest, the outbreak did not spread to any patients, nor were measles cases reported from the surrounding community. Given the high transmissibility of measles, the lack of transmission to patients may indicate that few patients were susceptible.

In China, vaccination coverage levels among children are high, and this may have prevented community spread. With a low incidence of measles, clinicians have little experience with the disease and may not recognize signs and symptoms of measles [1, 5]. Diagnosing measles in adults can be more difficult than in children because the rash and prodromal fever may be subtle, as described by Shakoor and colleagues [5]. Our survey showed that just over half of the health care workers had accurate knowledge about measles. In addition to knowledge about diagnosing measles, isolation and management of suspected cases is also important [6].

Measles vaccination coverage was low among the hospital’s health care workers, as less than half reported being vaccinated. Less than half believed measles to be a serious threat to health, and less than a third expressed willingness to get vaccinated. However, HCWs are at increased risk for acquiring infections, and they can act as vectors for transmission of measles. Vaccination is an effective means of preventing occupational exposure to measles from resulting in acquisition of measles. The United States Centers for Disease Control and Prevention recommends that all health care workers should be immune to measles. Many countries, including Belgium, Cyprus, Germany, France, Ireland, Italy, Lithuania, Luxemburg, Greece, Malta, Russia, Spain, Switzerland, Estonia, the United Kingdom [UK], Norway, and the Netherland recommend measles vaccination for all HCWs. Austria recommends vaccination for pediatricians [7–9], and Finland has a policy of mandatory measles vaccination of HCWs [10]. In 2015, China’s National Health and Family Planning Commission (NHFPC) recommended that health care workers under 50 years of age receive at least one MCV dose if they have unknown vaccination status and have not had measles ([2015]52). Hospital W had not implemented this recommendation prior to the outbreak.

According to the Chinese National Measles Surveillance guidelines [11], a patient with fever and rash that is accompanied with cough, coryza, or conjunctivitis should be reported though NNDRS. County CDC staff are responsible for conducting a case investigation and obtaining appropriate blood specimens. In this outbreak, reporting to NNDRS was delayed, which may have led to the spread of measles in the hospital.

Strengths of this investigation included that measles specialists interviewed all measles-confirmed individuals and conducted a hospital-wide search for additional cases. A weakness in the investigation was that we could not conduct active search for measles cases in community - instead relying on reporting to NNDRS for identification of community cases. Vaccination histories were obtained by self-report, which tends to overestimate coverage; thus, coverage among health care workers may have been lower than determined by the questionnaire.

## Conclusions

Low vaccination coverage among HCWs created the conditions that allowed this outbreak to happen, and delay in diagnosing measles and delayed reporting allowed the outbreak to continue. Providing measles vaccine to HCWs may have limited spread among HCWs.

We believe that our investigation supports some recommendations. First, the NHFPC recommendation to vaccinate health care workers should be fully implemented. Guidelines for implementation should be developed, and evaluation of hospital compliance with the recommendation should be conducted. Second, methods to improve vaccination rates under this policy should be identified and evaluated, including offerings vaccine, assessing HCW compliance, and assessing attitudes of hospital managers toward measles prevention. Third, training materials for health care workers should be developed and used to provide education about measles and measles prevention. Fourth, timely reporting through NNDRS needs to be reinforced through education and training. Fifth, hospitals infection control practices should include measles prevention and management.

## Abbreviations

CDC: Center for Disease Control and Prevention
HCW: Healthcare worker
MCV: Measles-containing vaccine
NHFPC: China’s National Health and Family Planning Commission
NNDRS: National Notifiable Disease Reporting System
SIA: Supplementary immunization activities
